# Medical Lectures

**Published:** 1851-11

**Authors:** 


### MEDICAL LECTURES.

The season for medical lectures is upon us, and most of the medical schools of the country have already commenced their preliminary courses of instruction.

The only important matter connected with this matter, beyond the statements of the annual announcement, is the return of Professor Silliman to the West. He comes back from his European travels, abundantly prepared to present before his classes all the improvements recently made in his department of medical science, a department that will have to be recognised by the practitioner of scientific medicine, as second to none in medical science, in qualifying men for the practice of medicine. There are few books of more vital importance to the practitioner than Fownes' Chemistry, and Bird on Urinary deposits. The paper we published some months ago, on Carbonagogue Medication, from the pen of Professor

Rogers, shows how admirably an intimate acquaintance with chemistry, may aid the physician in the diagnosis of disease, and in selecting the exact remedies for the diseased condition. B.

